# Medieval Roots of the Myth of Jewish Male Menstruation

**DOI:** 10.5041/RMMJ.10454

**Published:** 2021-10-25

**Authors:** Noga Roguin Maor, Ariel Roguin, Nathan Roguin

**Affiliations:** 1 Clalit Health Services, Haifa and Western Galilee District, Nahariya, Israel; 2 Department of Cardiology, Hillel Yaffe Medical Center, Hadera, Israel; 3 The Ruth & Bruce Rappaport Faculty of Medicine, Technion–Israel Institute of Technology, Haifa, Israel

**Keywords:** Anti-Semitism, Jewish, hemorrhoids, male, menstruation

## Abstract

The Jews in Western Europe during the middle ages were often perceived as distinct from other people not only in their religion, but also by virtue of peculiar physical characteristics. Male Jews were circumcised, which made them physically distinct in the sexual realm. They were believed to have a flux of blood due to hemorrhoids that was thought to more abound in Jews because they consumed salty foods and gross undigested blood, and were melancholic. By the late medieval and early modern periods, the male menstruation motif had become closely connected to the theory of the four humors and the balance between bodily fluids. Men in general were thought of as emitting extra heat, whereas women were considered to be physically cooler. While most men were generally able to reduce their heat naturally, there was a perception that womanish Jewish males were unable to do so, and thereby required “menstruation” (i.e. a literal discharge of blood) in order to achieve bodily equilibrium. The Jewish male image as having menses due to bleeding hemorrhoids was an anti-Semitic claim that had a religious explanation: Jews menstruated because they had been beaten in their hindquarters for having crucified Jesus Christ. This reflection is one of the first biological-racial motifs that were used by the Christians. Preceding this, anti-Semitic rationalizations were mostly religious. However, once these Christians mixed anti-Semitism with science, by emphasizing the metaphorical moral impurity of Jews, the subsequent belief that Jewish men “menstruated” developed—a belief that would have dire historical consequences for the Jewish communities of Europe until even the mid-twentieth century. This topic has direct applicability to current medical practice. The anti-Semitic perspective of Jewish male menstruation would never have taken hold if the medical community had not ignored the facts, and if the population in general had had a knowledge of the facts. In the same way, it is important for present-day scientists and healthcare professionals to understand thoroughly a topic and not to deliberately ignore the facts, which can affect professional and public thought, thereby leading to incorrect and at times immoral conclusions.

## BACKGROUND

The end of the thirteenth century and beginning of the fourteenth century were significant and challenging times for Jewish communities in Western Europe. King Philip the Fair expelled the Jews from Gascony (south-west of France) in 1288. Jews were expelled from England in 1290, and from France in 1306. Dissent to the inhumane national Jewish expulsions was rare. In the early fourteenth century, in a debate held at the Theological Faculty at the University of Paris on the topic entitled “Should Jews who have been expelled from one region be expelled from another?”, the sole dissenting voice was that of the Cistercian theologian, Jacques de Therines. It was within this tumultuous time and social atmosphere that the idea or myth of menstruation among Jewish males appeared and spread throughout Europe.^1^–^4^

Today menstruation is defined as a cyclical discharging of blood, secretions, and tissue debris from the uterus of non-pregnant breeding-age primate females at approximately monthly intervals. However, there is a lesser-known understanding of the term, which was used historically: menstruation referred to bleeding in general. This manuscript discusses this lesser-known definition, specifically in reference to men.

## FIRST REFERENCES TO JEWISH MALE MENSTRUATION

The earliest explicit source asserting that Jewish men menstruate appears in 1219. Jacques de Vitry (1160–1240) was a French canon regular who preached between 1211 and 1213 at the Albigensian Crusade and the Fifth Crusade in the East. In 1214 he was elected Bishop of Acre, in the Holy Land. In 1219 he began to write *Historia Orientalis*, a history of the Holy Land from the advent of Islam until the crusades of his own day. He wrote about the Jews: “… His blood be upon us ... have become unwarlike and weak as women, and it is said that they have a flux of blood every month ...”;^2^ de Vitry notes that his report was based on what others before him had already said.

This description led to two of the important elements in the Christian iconography of Jewish males: firstly they were depicted as being womanish, and secondly that they had a monthly flux of blood from their hinder parts as divine punishment. De Vitry also wrote that “The Jews had a trembling head, a quaking heart and perpetual anxiety and fearfulness—all signs of melancholia.”^2^

## THEOLOGICAL ASPECTS

In his seminal review, Hastings^3^ wrote that anal bleeding was thought to be the just punishment for verbal sins and that the myth’s formulation occurred in the context of Christ’s Passion and the punishment of Judas Iscariot, whose death included the rupture of his viscera through his anus. From there, the theory developed that anal bleeding could be a punishment for the denial of Christ’s divinity.

The Cistercian monk Caesarius of Heisterbach suggested in 1240 that Jewish males menstruated on a regular basis.^1^,^4^ He wrote that the Jews are “obstinate men, unwarlike even more than women, everywhere serfs, suffering from a flow every month.”^4^ In a thirteenth-century manuscript from the Cistercian abbey of Heiligenkreuz, in the Rhineland, the Jews were said to labor under some frailty, which is called a “flow of blood.”^4^

Mixing anti-Semitism with science, and attempting to demonstrate the racial impurity of Jews, scholars claimed that Jewish males menstruated to rid the body of unclean blood. Although these accounts came from a place of prejudice, they nonetheless demonstrate the prevailing early modern belief in male menstruation and its role in ridding the body of unwanted blood.

## MEDIEVAL UNDERSTANDING OF SEX AND MENSTRUATION

The medieval understanding of sex was grounded in the writings of the Greek philosopher Aristotle and the Greek physician Galen. They had postulated a structural homology between the sexual organs of men and women, whereby they were basically the same, except that those of men lay outside the body while those of women lay inside and were reversed.

Bleedings in males, whether pathological or induced by clinical practices, was thought to rid the body of excess and potentially dangerous blood. To understand these observations, the factors that shaped medical thought during this period merit a closer look.

### Humorism

The idea that a balance of humors achieves good health descended from the enduring influence of Galen and directly led to the ubiquitous endorsement of bleeding. Humorism, which had originated with Hippocrates and was later taken up by Galen, suggested the existence of four bodily fluids or humors: black bile, yellow bile, phlegm, and blood. If an imbalance occurred, illness ensued. Therefore, to restore the health of the patient, blood was drawn from the body in an effort to achieve equilibrium. Menstruation was often conceptualized as a similar but natural means of ridding the body of excess blood. However, the question of why females, and not males, menstruated remained.

### Heat Dissipation and Bleeding

Explanations for menstruation were rooted in beliefs concerning male heat and female coolness. Since it was believed that males generated a significant amount of heat (another principle derived from Galen), men would thus be able to burn off the excess materials that women could only be rid of during menstruation. Since females were considered cooler, it was believed they were unable to burn excess blood. However, certain males did not generate sufficient heat to return the humors to a desirable balance. Therefore, it was necessary for them to remove the excess blood through other means, such as nosebleeds.

### The “Other”

Even though Christians in Europe did not observe purity rituals as did Muslims and Jews, they did define themselves against the “other” using the rhetoric of impurity. Medieval Christian men believed that they were intellectually and biologically superior to women.

Masculinity connoted aggressive behavior and sexual assertiveness, whereas femininity connoted submissiveness and powerlessness.^5^ Illness was considered a mark of femininity in males.

Characteristics such as timidity and pallor were understood by medieval authors to be womanish, and they clearly perceived that, just as women may exhibit masculine traits on occasion, so too men could display the characteristic traits of women.^5^ Sexual characterization became a powerful polemic tool used by Christians to feminize Muslims and Jews. In the Castilian Reconquista epic, *Cantar de Mio Cid*, for example, a male Jew was given the female name Raquel, to depict a Jew as feminine, making him weak.^6^

## HEMORRHOIDS IN JEWISH MALES

The most eminent German philosopher and theologian of the middle Ages, and the founder of the scientific tendency within the Catholic Dominican order, was Albertus Magnus (also known as Albert the Great [1193–1280]).^1^,^2^ He was recognized by the Catholic Church as one of the 36 Doctors of the Church (in Latin: *Doctoris Ecclesiae Universalis*), a title given to saints recognized as having made a significant contribution to theology or doctrine through their research, study, or writing.

He was the first to comment on virtually all of Aristotle’s writings, thus making them accessible to wider academic debate. His study of Aristotle contributed to his perspectives on the teachings of Muslim academics, notably Avicenna and Averroes, and the Jewish masters Maimonides and Avicebron (Solomon Ibn Gabirol). In particular, Albert wrote about melancholia and that Jews were naturally disposed to envy, extreme hairiness, and luxuria (increased sexual desire). With regard to hemorrhoids, he wrote: “Hemorrhoids are caused as a purging of melancholic blood from the veins around the anus … this happened, mostly to those who lives of gross food and salted food, such as the Jews, and this happened according to nature.”^4^

On the other hand, the Persian physician Avicenna (Ibn Sina) (980–1037), author of the famous *Canon of Medicine*, described bleeding hemorrhoids and their treatment, but did not indicate that this condition was more common in Jews.^7^ In his *Treatise on Hemorrhoids*,^8^ Maimonides (1138–1204) connected hemorrhoids with black bile and melancholic patients, but did not note them as being more common among Jewish patients.

Around 1300, in a debate in the Paris University, Henry of Brussels and Henry the German concluded that the Jews have a flux of blood due to hemorrhoids caused by gross indigested blood which nature purges, and that this abounds more in Jews because they, for the most part, are melancholic. They added that Jews naturally withdraw themselves from society and from being connected to others, that they are more likely to be cut off or live in solitary places, and that they are pallid and timid. They went on to claim the Jews consumed roasted or fried foods rather than boiled, and that such foods are difficult to digest; that they do not drink wine; and that they have no or very little blood-letting, resulting in them emitting blood through pores.^1^,^2^

Bernard de Gordon (1270–1330) was a medical professor from the Montpellier University. In his medical book, *Lilium Medicinae*, he explained why Jews suffered from hemorrhoids:

The Jews suffer an immoderate flow of blood from hemorrhoids, for three reasons: generally, because they are in idleness, and for that reason the melancholic superfluities are gathered. Second, they are generally in fear and anxiety, and for this reason melancholic blood is increased, according to this [saying] of Hippocrates: “Fear and timidity, if they have a lot of time [to work], generate the melancholic humor.” Third, this occurs as a divine punishment, according to [the text], “And he struck them in their posteriors and gave them over to perpetual opprobrium.”^3^

De Gordon’s first two arguments justified hemorrhoids using humoral medicine, stating that Jews produced a surplus of the humor melancholy, a surplus or humoral imbalance, and that these occurred because the Jews were continually lazy and in fear, and building a human body/politic equation into his explanation. For Gordon, the humoral explanation—impure blood due to the presence of melancholic humor—naturally gave way to the third, religious, explanation: Jews menstruated not only because they have a sedentary lifestyle but because they crucified Christ.

This ideological jump, from an explanation that had to do with the inner workings of the body to a religious-racial one, makes sense when one remembers the way that Gordon and other medieval scholars would have understood the nature of disease. Gordon naturally combined an explanation of how the diseased blood flow occurred within the individual body with the notion that the Jews received the illness because of a divine punishment, an explanation that reflected the fear that the heresy of Judaism was a threat of pollution to the body politic.^3^–^6^

## ANTI-SEMITIC CONSEQUENCES

The belief that Jews suffered from moral impurity, physically manifesting as the Jewish male flux, had powerful consequences for Jewish communities throughout Europe.^5^–^10^ The Fourth Lateran Council confined Jews to their homes during Holy Week in order to protect Christian children from bloodthirsty Jews. In 1235, Christian religious leaders cited the medicinal value of Christian blood as proof for the blood libel at Fulda. Jews were expelled from Thuringia in 1400 because of “their need for human blood to heal a wound that flowed in them perpetually.”^5^ And in Tyrnau in 1494, twelve Jewish men and two women were accused of killing a Christian child: they were forced to confess that “men and women among them suffer equally from menstruation … The blood of a Christian is the specific medicine for it, when drunk”; all fourteen were burned.^5^

References to Jewish male menstruation have been found in Germany in 1614 and England in 1649. One sixteenth-century English author writes that “Jews, men, as well as females, are punished *cursu menstruo sanguinis*, with a very frequent blood-fluxe.”^9^

These medieval myths persisted beyond the middle ages, especially in Spain. In 1632 Juan de Quiniones and Juan de la Huerta, both royal Spanish physicians, asserted that Jews suffered from a permanent menstruation, a blood flow from their lower regions, the substance itself being impure blood.^2^,^7^

Pedro Aznar Cardona wrote in 1612: “many of them [Jews] are born with lizard tails and suffer ignominious and uncomfortable hemorrhoids.”^2^ Quiniones and Huerta gave a religious explanation: “the Jewish illness was due to their denial of Christ.”^2^ Torrejoncillo wrote: “The day of crucifixion, the spilling of Christ’s blood was the day that the Jews suffered their body malady. On Good Friday, all Jewish men and women experience a flow of blood.”^8^

In Spain, legal language was used that sought to exclude people of “impure blood.” Doctor Quiniones wrote his medical tract to the Grand Inquisitor Fray Antonio de Sotamayor and suggested a way to recognize people belonging to the Jewish race. Doctor Huerta even advocated adopting the idea that prohibited anyone of impure blood ancestry from entering the medical profession.

However, one Jewish doctor eventually did speak out—albeit outside of Spain. Isaac Cardoso was born in Portugal in 1603 to Marrano Jews who had converted or been forced to convert to Christianity, yet continued to practice Judaism in secret. Cardoso left Spain, probably to escape from the Inquisition, and moved to Venice, Italy. He returned to Judaism and later settled in Verona. He wrote a book refuting the assumption of Jewish hemorrhoids or menstruation that was common in seventeenth-century Spain. In his monograph, *Las excelencias de los Hebreos* (The excellence of the Hebrews), he refuted all the false accusations. The monograph was printed in Amsterdam in 1679.^9^–^11^

### Later Centuries

By the turn of the twentieth century, the old claim of Jewish male menses was closely tied to the new image of the weak and pale Jewish male and was well on its way to being transformed into a racial gender theory. Austrian and Germanic culture in the late 1800s and early 1900s valued physical appearance. It is therefore unsurprising that in early twentieth-century in Germany, the menstruating Jewish male appears prominently in Nazi-era anti-Semitic literature.

This personification, which had persisted for over 700 years, was harmful not only in its caricatures of Jewish men, but also because it was used to promote anti-Semitism. The bias against Jewish men and all women went hand-in-hand for some 700 years and suggests that these two forms of discrimination were closely connected, historically and perhaps even conceptually. Woman, already considered the “Other” or outsider by many societies throughout history, was the perfect tool with which Christians could stigmatize the Jew as the ultimate religious and ethnic outsider.

## CONCLUSIONS

In summary, we present an intriguing charge by medieval Christians that Jewish men menstruated. This was explained as part of the punishment due to their responsibility for the death of Jesus. Jews menstruated because they had been beaten in their hindquarters for having crucified Christ, and had hemorrhoids due to dietary habits and melancholia.

This particular anti-Semitic libel, which mixed anti-Semitism with science, seems to have been widespread throughout early modern Western Europe, not only within clerical circles, but also within secular and popular society.^4^ Evolving from roots at least as far back as late antiquity, it arrived in its “mature” form in thirteenth-century texts. This belief would have dire historical consequences for the Jewish communities of Europe for centuries.

This image is one of the first biological-racial motifs that were used by the Christians. Preceding this, anti-Semitic rationalizations were mostly religious. Contemporary conceptions of embodiment and gender are radically different from medieval thought on the subject. Lack of knowledge at that time, or a deliberate refusal on the part of the doctors and clergy to see the facts, led to flawed theories and education based on falsehoods. Today, we have seen dramatic improvements in Jewish–Christian relations, and the male menstruation myth no longer exists. Still, even if largely forgotten, it remains an important false fabrication to remember: telling the story is a crucial reminder to all of the importance of how facts are approached, how to interpret evidence, and the negative impact of preconceptions on humanity, regardless of sex or religion.
